# Equitable tuberculosis care in the North West of England: analysis of tuberculosis cohort review data

**DOI:** 10.5588/ijtld.15.0772

**Published:** 2016-06-01

**Authors:** P. MacPherson, S. B. Squire, P. Cleary, S. Davies, C. Wake, K. Dee, J. Walker, S. Farrow, P. McMaster, M. Woodhead, D. J. Sloan, on behalf of the North West TB Audit Steering Committee

**Affiliations:** * Department of Public Health and Policy, University of Liverpool, Liverpool; † Department of Clinical Sciences, Liverpool School of Tropical Medicine, Liverpool; ‡ North West Public Health England Centre, Liverpool, Liverpool; § Centre for Applied Health Research & Delivery, Liverpool School of Tropical Medicine, Liverpool; ¶ Public Health England Field Epidemiology Service North West, Liverpool; # TB Section, Liverpool Community Health, Liverpool; ** Department of TB Services, Pennine Acute Hospitals NHS Trust, Oldham; †† North Manchester General Hospital, Manchester; ‡‡ Department of Respiratory Medicine, Central Manchester University Hospitals NHS Foundation Trust, Manchester; §§ Faculty of Medical and Human Sciences, University of Manchester, Manchester, UK

**Keywords:** tuberculosis, socio-economic deprivation, TB cohort review, patient-centred care, England

## Abstract

BACKGROUND: In the United Kingdom, tuberculosis (TB) predominantly affects the most deprived populations, yet the extent to which deprivation affects TB care outcomes is unknown.

METHODS: Since 2011, the North West TB Cohort Audit collaboration has undertaken quarterly reviews of outcomes against consensus-defined care standard indicators for all individuals notified with TB. We investigated associations between adverse TB care outcomes and Index of Multiple Deprivation (IMD) 2010 scores measured at lower super output area of residence using logistic regression models.

RESULTS: Of 1831 individuals notified with TB between 2011 and 2014, 62% (1131/1831) came from the most deprived national quintile areas. In single variable analysis, greater deprivation was significantly associated with increased likelihood of the completion of a standardised risk assessment (OR 2.99, 95%CI 5.27–19.65) and offer of a human immunodeficiency virus test (OR 1.72, 95%CI 1.10–2.62). In multivariable analysis, there were no significant associations.

CONCLUSIONS: TB patients in the most deprived areas had similar care indicators across a range of standards to those of individuals living in the more affluent areas, suggesting that the delivery of TB care in the North West of England is equitable. The extent to which the cohort review process contributes to, and sustains, this standard of care deserves further study.

## RESUME

CADRE : Au Royaume-Uni, la tuberculose (TB) affecte de façon prédominante les populations les plus déshéritées, mais on ne sait pas jusqu'à quel point la pauvreté affecte les résultats de la prise en charge de la TB.

MÉTHODES : Depuis 2011, la collaboration du North West TB Cohort Audit a entrepris une revue trimestrielle des résultats du traitement par rapport aux indicateurs standard de soins définis par consensus pour tous les individus déclarés tuberculeux. Nous avons recherché des associations entre un mauvais résultat du traitement de la TB et les scores de l'Index de Privations Multiples 2010 mesurés aux plus bas des zones statistiques du lieu de résidence grâce à des modèles de régression logistique.

RÉSULTATS : Sur 1831 individus déclarés tuberculeux entre 2011 et 2014, 62% (1131/1831) venaient des zones les plus déshéritées du quintile national. En analyse à une seule variable, une plus grande pauvreté a été significativement associée à une probabilité accrue de remplissage d'une évaluation de risque standardisé (OR 2,99 ; IC95% 5,27–19,65) et d'offre de test pour le virus de l'immunodéficience humaine (OR 1,72 ; IC95% 1,10–2,62). En analyse multivariée, il n'y a pas eu d'association significative.

CONCLUSION : Les patients TB des zones les plus déshéritées ont eu des indicateurs de soins similaires dans la limite des standards aux personnes vivant dans des zones plus aisées, suggérant que la prestation de soins de TB dans le Nord Ouest de l'Angleterre est équitable. D'autres études sont requises pour savoir jusqu'à quel point le processus de revue de la cohorte contribue à ce niveau de soins et le soutient.

## RESUMEN

MARCO DE REFERENCIA: En el Reino Unido, la tuberculosis (TB) afecta sobre todo a las poblaciones más desfavorecidas, pero se desconoce la magnitud del efecto de la privación en el desenlace clínico de la TB.

MÉTODO: Desde el 2011, la iniciativa de cooperación ‘North West TB Cohort Audit’ emprendió un examen trimestral de los desenlaces clínicos con respecto a una serie de indicadores normalizados definidos unánimemente, en todas las personas notificadas con TB. En el presente artículo, mediante modelos de regresión logística, se investigaron las asociaciones entre los desenlaces desfavorables de la atención de la TB y las puntuaciones del índice de privación múltiple del 2010, medido en las zonas de residencia según una unidad geográfica para áreas estadísticas pequeñas.

RESULTADOS: De los 1831 casos de TB notificados del 2011 y el 2014, el 62% (1131/1831) provenía de zonas de los quintiles más desfavorecidos del país. En un análisis monofactorial, una mayor privación se asocióde manera significativa con una mayor probabilidad de compleción de una evaluación normalizada de riesgos (OR 2,99; IC95% 5,27–19,65) y con la propuesta de una prueba diagnóstica de la infección por el virus de la inmunodeficiencia humana (OR 1,72; IC95% 1,10–2.62). El análisis multifactorial no puso en evidencia asociaciones significativas.

CONCLUSIÓN: Los indicadores de la calidad de atención de los pacientes con TB en las zonas más desfavorecidas fueron equivalentes a los indicadores de las personas residentes en zonas más favorecidas, con respecto a toda una serie de normas; este resultado indica que la prestación de la atención de la TB en el noroccidente de Inglaterra es equitativa. En futuros estudios se deberá evaluar en qué medida este procedimiento de examen de cohortes contribuye a la calidad de la atención prestada y a su mantenimiento.

HISTORICALLY, THE RISK of tuberculosis (TB) disease has been strongly associated with poverty. Indeed, the large gains in poverty reduction in Europe in the early and mid-twentieth centuries are recognised to be major contributors to the dramatically reduced TB incidence rates over the same period.^1^ In recent years, however, rates of absolute and relative poverty among people in England have sharply increased, mostly driven by increasing income inequality and disproportionate cuts in welfare provision and local services in the poorest communities.^2^,^3^

The North West of England, which contains some of the poorest cities and communities in England, has been disproportionately affected by inequality.^3^ Indicators for economic development, standards of living, early childhood development and overall population health are significantly worse in the northwest compared to the south of England.^3^

Public Health England (London, UK) has declared TB to be a national public health priority,^4^ recognising that incidence rates are substantially higher than in most other Western European countries,^5^ and, in absolute numbers, nearly on a par with the United States, which has a five-fold greater population.^5^ Individuals living in the most socio-economically deprived communities in England have the highest TB incidence rates in the country, a phenomenon that is perpetuated by interactions with risk factors for TB, including alcoholism, drug use, incarceration, homelessness and migration from high TB incidence countries.^6^

However, the effect of socio-economic deprivation on individuals' ability to access TB care in a timely manner and achieve a successful treatment outcome is not well understood.^7^ Studies among individuals with human immunodeficiency virus (HIV) infection^8^ and cystic fibrosis^9^ show that socio-economic deprivation is a substantial contributor to delayed treatment and adverse treatment outcomes.

Formal TB cohort review programmes were pioneered as a strategic tool to improve TB care and prevention in Tanzania.^10^ Following successful implementation in New York City, NY, USA, in the 1990s,^11^,^12^ TB cohort review was first implemented in the United Kingdom in London,^13^ from where the great majority of UK TB cases are notified.^6^ However, the North West of England represents the largest footprint where TB cohort review has been undertaken, and covers a diverse range of populations and geographical settings.

The objective of the present study was to investigate the effect of socio-economic deprivation on care outcomes among individuals notified with TB in the North West of England using robust, prospectively collected data from quarterly TB cohort reviews and Public Health England surveillance systems. A greater understanding of the effects of socio-economic deprivation on care outcomes among individuals from one of the poorest regions of the country could drive efforts to improve equitable and effective care, ultimately improving TB control.

## METHODS

### Study site and population

The North West of England covers a population of approximately 7 million living in a wide variety of settings, including major urban cities such as Manchester, Liverpool, Blackburn and Preston, as well as more rural areas in Cumbria (Figure). The population is served by 23 local authorities and by three Public Health England Centres (Cheshire and Merseyside, Cumbria and Lancashire, and Greater Manchester, recently merged to form the North West Public Health England Centre) that provide health protection services to their local populations in collaboration with acute hospital care trusts. In England, TB cases are statutorily notified to Public Health England.

**Figure:**
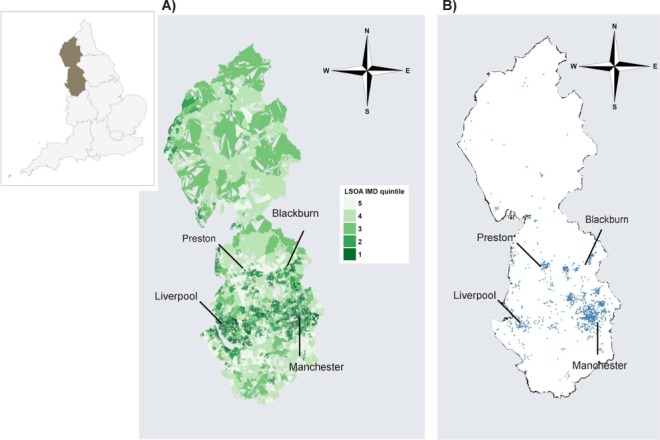
TB cases in relation to LSOA IMD 2010 rank quintile, North West England, 2011–2014. **A)** Choroplethic map showing LSOA IMD 2010 rank quintile (1 = most deprived; 5 = least deprived). **B)** Tuberculosis cases (2011–2014) plotted to centroid of 2011 LSOA. Shapefiles for maps from the UK Office for National Statistics (ons.gov.uk). Insert shows North West England (highlighted). LSOA = lower super output areas; IMD = Index of Multiple Deprivation. This image can be viewed online in colour at http://www.ingentaconnect.com/content/iuatld/ijtld/2016/00000020/00000006/art00012.

The North West TB Cohort Audit (NWTBCA) was established in April 2012 as a workstream of a regional initiative to improve TB control. The objectives of cohort review are to collaboratively improve the implementation of comprehensive case management and standardise care for all TB patients in the North West of England by evaluating cohort review data and identifying appropriate areas for intervention. TB cohort reviews are held quarterly and are attended by TB nurses and physicians, epidemiologists, public health specialists and analysts. During TB cohort review meetings, the TB diagnostic and care outcomes of all newly notified cases of TB identified through the Public Health England Enhanced TB Surveillance (ETS) database are reviewed anonymously against NWTBCA consensus-defined standard of care indicators (Table 1). Standard of care indicators relate to either quality of care provided during TB diagnosis, treatment and screening of contacts, or to care outcomes, and are intended to be targets that are routinely assessed during cohort review meetings.

**Table 1:**
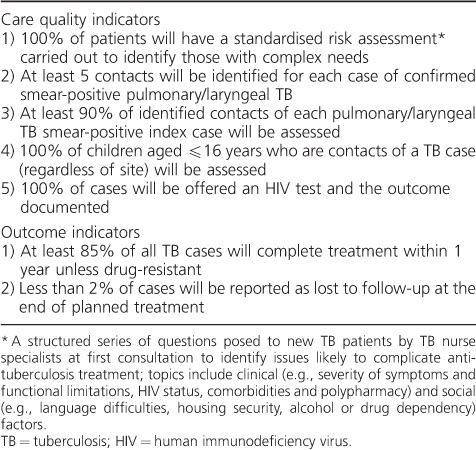
North West TB Cohort Audit standard of care indicators evaluated in this study

### Data collection and analysis

We used an anonymised data set comprising TB cases from North West England notified to Public Health England's ETS database and reviewed at NWTBCA reviews between 2011 and 2014. No restrictions were placed on inclusion for this analysis.

English lower super output areas (LSOAs) are small, census-defined statistical geographical boundaries comprising approximately 1500 people. We used England LSOAs as defined by the Office of National Statistics, Newport, UK, in 2011. Each notified TB case was assigned to an LSOA on the basis of postcode of primary residence at the time of notification to Public Health England, with a consistency check to ensure that all assigned LSOAs were located within the North West England geographical footprint.

The main exposure of interest was socio-economic deprivation, measured using the English Indices of Multiple Deprivation (IMD) at the level of TB cases' LSOA of residence. English Indices of Deprivation are constructed nationally using census information and other local government administrative data. They include 38 indicators in seven domains: income, employment, health and disability, education skills and training, barriers to housing and other services, crime, and living environment. Overall IMD scores have been constructed for each LSOA in England by ranking aggregated weighted domain scores. We constructed quintiles of national IMD rank scores (ranging from 1, most deprived to 5, least deprived) and linked these to TB cases' LSOA of residence at the time of notification. Where individuals had no postcode of residence and were recorded as homeless, we assigned them to the most deprived quintile.

Standardised demographic, clinical and social risk factor characters are routinely recorded by TB nurses during clinical assessments and entered into the ETS database hosted by Public Health England using a secure web portal. Additional data on outcomes and other risk factors are obtained at TB cohort reviews. The combined data were linked to participants' IMD quintile group using the postcode. Where data on the presence or absence of a social risk factor (injecting drug use, homelessness, incarceration, alcoholism) were not recorded, we assumed that the risk factor was not present. Characteristics were compared across IMD quintile groups using χ^2^ and Fisher's exact tests.

NWTBCA consensus-defined standard of care indicators were dichotomised, with TB cases who were recorded in the cohort review database as having met the outcome classified as being a ‘success’. Single variable logistic regression models were constructed to investigate associations between having experienced a successful care outcome, IMD rank quintile group and other characteristics. Multivariable models were adjusted a priori for age group, sex, UK-born status and the presence of any social risk factor (injecting drug use, alcoholism, incarceration or homelessness). Where date of symptom onset was not recorded, individuals were excluded from analysis. R version 3.1.3 (R Foundation for Statistical Computing, Vienna, Austria) was used for all analysis.

### Ethical considerations

As the study used anonymised routinely collected public health surveillance data, individual participant assent was not sought. The North West TB Summit Steering Committee formally reviewed and approved the study protocol.

## RESULTS

### Characteristics of TB cases

Between 2011 and 2014, 1932 TB cases were notified to Public Health England and included in this analysis. The postcode of residence of 101 (5.2%) participants could not be linked to IMD scores, leaving 1831 participants in the final data set.

TB cases were predominantly clustered in the major urban centres of the North West of England, with the majority of cases residing in Manchester, Blackburn and Liverpool (Figure). The distribution of TB cases was strongly related to national IMD rank quintile: 1132 of 1831 (61.8%) cases resided in LSOAs in the most deprived national IMD rank quintile compared to only 106 (5.8%) in the least deprived quintile (Table 2).

**Table 2:**
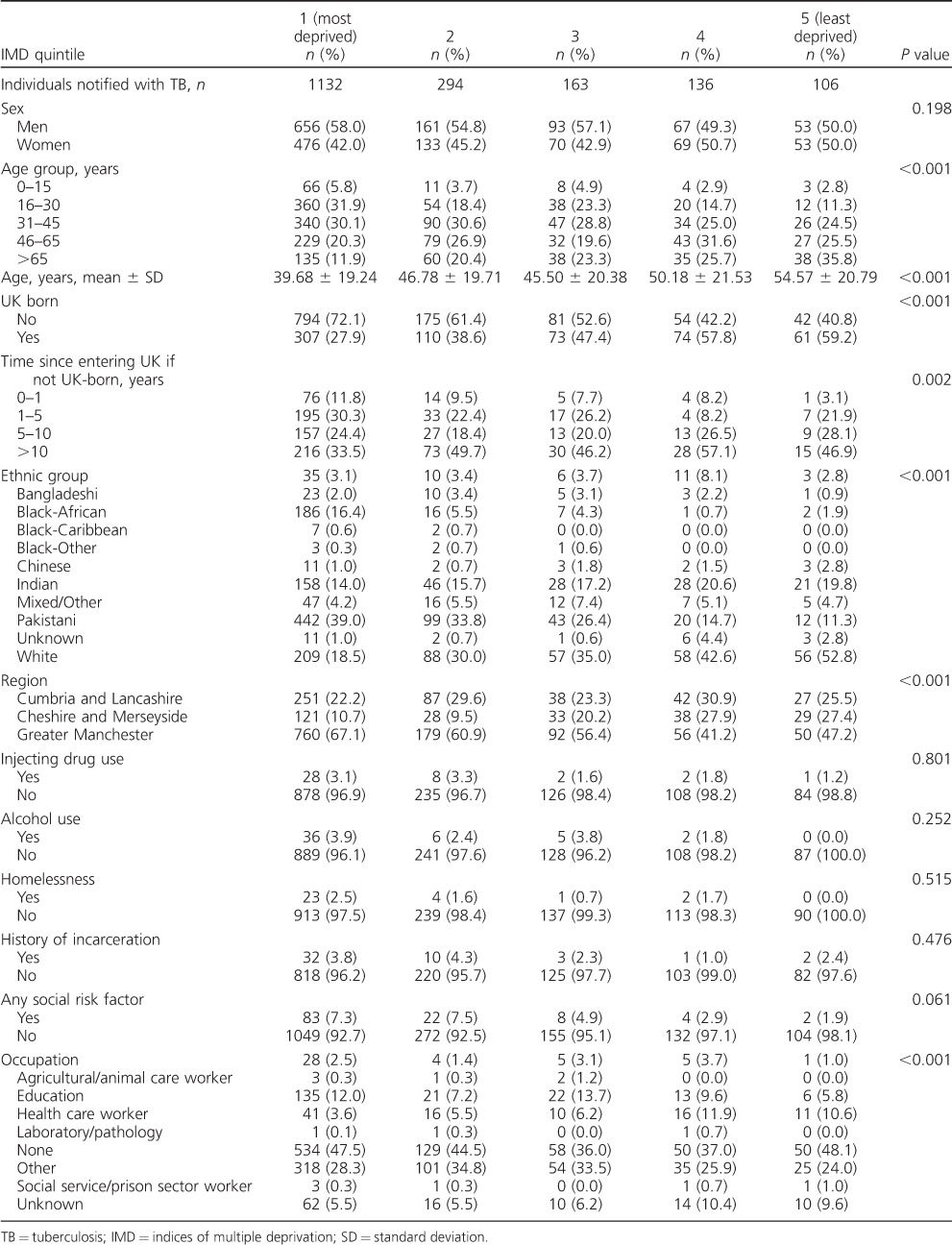
Characteristics of notified TB cases in North West England, 2011–2014, by English IMD rank quintile

Compared to individuals in less deprived groups, TB cases from the most deprived socio-economic quintile were more likely to be younger (*P* < 0.001), born outside the UK (*P* < 0.001), in a minority ethnic group (*P* < 0.001) and have a social risk factor for TB (borderline association, *P* = 0.061).

### TB standard of care indicators

Overall, when assessed against consensus-defined standard of care indicators, outcomes were consistent between socio-economic groups (Table 3). In each socio-economic group, >90% of TB cases had a standardised risk assessment completed, 79–85% of TB cases had ⩾90% of close contacts evaluated for TB, >95% of child contacts were assessed, 67–78% were offered an HIV test, <1% were lost to follow-up, and >80% had completed anti-tuberculosis treatment at 12 months after diagnosis. However, outcomes of identification of at least five contacts per TB case were less consistently met, with only 10–22% of TB cases across socio-economic groups meeting this standard.

**Table 3:**
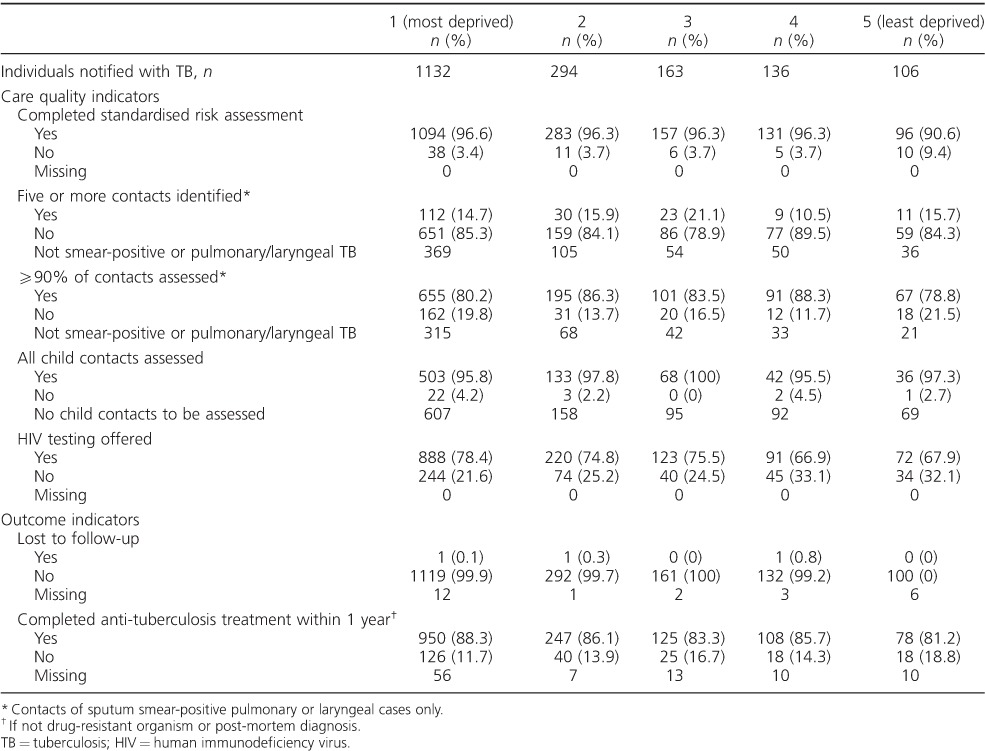
TB care indicators, North West England, 2011–2014

### Associations between deprivation and TB care standard of care indicators

On single variable analysis (Table 4), TB cases in the most socio-economically deprived group were significantly more likely to have a standardised risk assessment completed than TB cases in the least socio-economically deprived group (odds ratio [OR] 2.99, 95% confidence interval [CI] 5.27–19.65). Individuals in the most deprived group were also significantly more likely to be offered an HIV test (OR 1.72, 95%CI 1.10–2.62). There were no other significant associations between socio-economic groups and TB care outcomes.

**Table 4:**
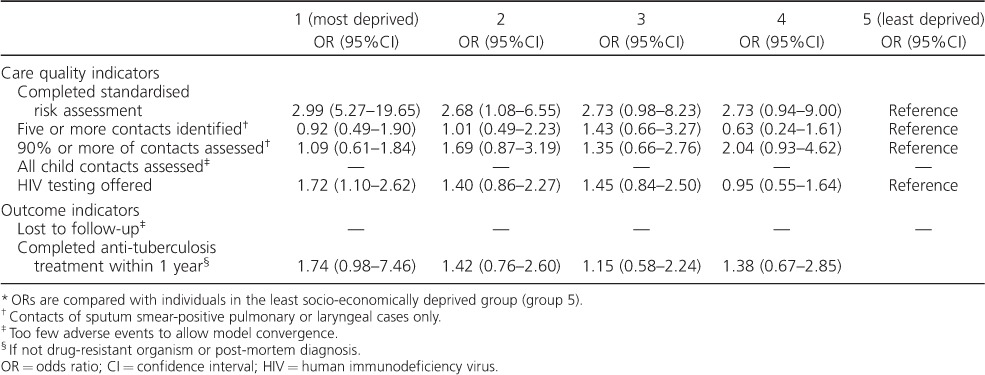
Single variable associations between socio-economic deprivation group and tuberculosis care indicators, North West England^*^

After adjustment for sex, age group, UK-born status and the presence of any social risk factors, no significant associations remained between socioeconomic group and TB standard of care indicators (Table 5).

**Table 5:**
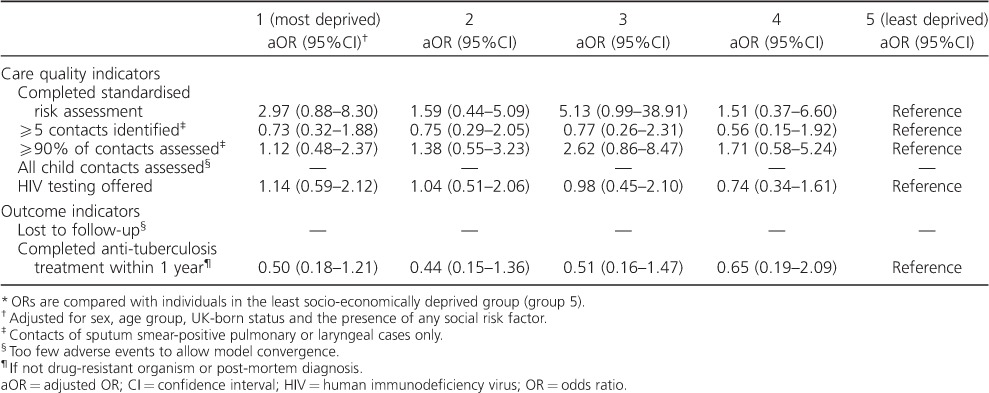
Multivariable associations between socio-economic deprivation group and tuberculosis care indicators, North West England^*^

## DISCUSSION

The main findings of this study were that although socio-economic deprivation was common among TB cases from the North West of England, TB patients in the most deprived group had similar care indicators to those of more affluent individuals across a range of TB quality and outcomes indicators, suggesting that access to and delivery of TB care through the National Health Service in the North West of England is equitable. Performance against consensus-defined care standards was consistent across all socio-economic groups over a period of 3 years. The contribution of the NWTBCA process to these impressive achievements requires further assessment.

In the TB cohort review process, a multidisciplinary team reviews the quality of care indicators for all notified TB cases for a defined geographic area, with the following objectives: ensuring that comprehensive, patient-centred cared is delivered, systematically identifying areas where care could be improved through auditing care outcomes against predefined standards in a timely fashion, and providing a forum for education and training for professionals and patient groups involved in delivering TB services. A central principle of the NWTBCA is to ensure that care delivered to TB patients is of high quality and is equitable. To our knowledge, this study represents the first attempt to quantify the effects of socio-economic deprivation on TB care outcomes using robust, validated exposure data and consensus-agreed outcome indicators. Given that data on socio-economic deprivation at the LSOA-level are made freely available by the UK National Office of Statistics, this analysis could be repeated by other regions undertaking cohort review, allowing comparison, identification of outliers requiring improvement or instances of outstanding best practice. Such analyses, if undertaken routinely, could drive improvements in TB care and prevention, a central objective of the new comprehensive UK TB strategy.^4^

The main finding that there were no significant differences in TB care indicators when individuals in different socio-economic deprivation quintiles were compared provides reassurance that the additional resources required by individuals with greater deprivation are necessary and justified. For example, non-UK-born individuals (which will include new arrivals into the United Kingdom, people who have entered the United Kingdom a number of decades before TB diagnosis and a smaller number of asylum-seekers and refugees) are overrepresented in the most deprived socio-economic groups,^14^ and frequently require substantial additional support, including translation, TB nurse-provided directly observed treatment and the support of social services and third sector organisations.^15^ Similarly, individuals in groups at risk for TB, including injecting drug users, homeless people, people with alcoholism and those who have been incarcerated, have previously been found to have high rates of loss to follow-up and worse treatment outcomes.^16^ In this population, these specific social risk factors for TB were found to be uncommon, ranging from 1.9% in the least deprived group to 7.3% in the most deprived group, and were not significantly associated with adverse TB care indicators on single variable analysis, although, due to the small numbers, this analysis is likely to be underpowered.

Individuals in the most deprived group did not fare significantly worse than those in less deprived groups. Although this analysis cannot causally attribute successful care outcomes to the intensified support provided to these groups, or to the effect of the TB cohort review itself, the growing body of evidence of the individual and public health benefits of TB cohort reviews means that further studies using a wider range of research methods (including qualitative studies, programmatic analysis and health needs assessments) would be justified.

In the absence of easily accessible and well-validated individual-level indicators of socio-economic deprivation, the development of the English IMDs, and other geospatially-defined measures such as the Townsend Deprivation Index and Carstairs Index, have been a major advance in public health and epidemiology, helping to explain how health and care are inequitably distributed in the UK population. Similar indices of deprivation have been validated in a number of other European countries and could be used to understand analyses comparable to ours, but have yet to be developed for the majority of high TB burden countries where the need is likely to be greatest.

Previous studies have shown that individuals from the United Kingdom's most socio-economically deprived groups have disproportionately worse access to care and outcomes for many conditions, including sexually transmitted diseases.^17^ Our finding that the most deprived TB patients were not significantly disadvantaged in care is therefore initially surprising. However, well-established recognition of the associations between poverty and TB has led North West TB programmes to focus on the needs of deprived populations, with enhanced case management strategies specifically designed to support vulnerable individuals. This approach may not have been so extensively integrated into the management of other diseases, and may go some way to bridging the inequality gap.

Although based on data from a robust national surveillance system and from TB cohort reviews, and despite the use of established and validated methods for categorising participants into socioeconomic deprivation groups, our analysis has a number of limitations. Because of the ecological study design, no causal relationship between socioeconomic deprivation and outcomes can be established. Socio-economic deprivation was measured at the LSOA level, in which indicators are aggregated over approximately 1500 people. Given that TB is often clustered among certain social groups (e.g., household members), alternative measures of deprivation (e.g., evaluated at household or individual level) may have been more valid, but would have required substantially greater study resources and the development and validation of new indices. To maximise the use of available data, we categorised homeless individuals into the most socio-economically deprived category; this may have resulted in misclassification of exposure status. Most adverse outcomes were experienced infrequently by individuals in each socio-economic deprivation group, meaning that CIs are wide; a study conducted in a larger geographical area (e.g., national) or for a longer period of time would allow more precise estimates of association. We did not examine outcomes such as mortality, morbidity, treatment delay and participant satisfaction or quality of life. Finally, there may have been potential for observer bias in recording outcomes, although this is likely to be mitigated by the regular and objective cohort review process.

In conclusion, this study, the largest of its kind to date, has found no significant association between socio-economic deprivation and worse TB care indicators. TB cohort review, such as implemented in the North West of England, has the potential to support the delivery of effective, equitable TB services, and the contribution of cohort reviews to success should be examined further.
